# Establishing a Community Foundation for Dementia Education, Engagement and Action among Black Older Adults

**DOI:** 10.1002/alz70858_103152

**Published:** 2025-12-25

**Authors:** Elena Flores, Jamie Mitchell

**Affiliations:** ^1^ University of Michigan, Ann Arbor, MI, USA

## Abstract

**Background:**

The Healthier Black Elders (HBE) program in Flint, MI established to advance health research engagement among Black older adults. Black adults are twice as likely to develop Alzheimer's disease and Alzheimer's disease related dementias (AD/ADRD) than their White counterparts. The HBE Community advisory board partnered with local research and aging services stakeholders to ascertain whether Black older adults in Flint, MI identify a need for tailored AD/ADRD educational programming, access to cognitive assessments and research studies on cognitive health.

**Method:**

Sixty (60) community‐dwelling participants (Mean age 67, 68% women) were recruited from the existing participant research pool of Black of older adults (*N* = 400, age 55+) overseen by the community advisory board of HBE Flint program. Six focus groups were conducted in a local senior center and facilitated by a trained community outreach specialist and research assistant. Focus groups were audio recorded and transcribed without identifying information. Participants were provided lunch but no incentives. A semi‐structured interview protocol focused on participants’ familiarity with AD/ADRD, stigma and concerns, interest in education, interest in and experience with cognitive assessments and research participation; as well as desired program components. Two study staff uninvolved with data collection conducted multi‐stage qualitative coding using a grounded theory approach; identifying key themes.

**Result:**

Key themes revealed a lack of health literacy about cognitive aging and perceptions of social stigma surrounding cognitive impairment. Participants were specifically interested in both resources for caregiving as well as how to prepare themselves for impacts to financial, health, and daily living should they be diagnosed with AD/ADRD. Participants expressed that any community‐focused curriculum should address skills for voicing concerns about cognitive changes to a healthcare provider and among family members. Finally, participants were broadly interested in and open to participating in free AD/ADRD cognitive assessments that provided actionable information; many participants were familiar with aging research and enthusiastic about participating in studies vetted by HBE or other trusted community entities.

**Conclusion:**

Black older adults in Flint welcome knowledge, programming, and research engagement on AD/ADRD; stakeholders should work with the community to address cognitive aging needs.

